# Bioactivation of Isoxazole-Containing Bromodomain and Extra-Terminal Domain (BET) Inhibitors

**DOI:** 10.3390/metabo11060390

**Published:** 2021-06-15

**Authors:** Noah R. Flynn, Michael D. Ward, Mary A. Schleiff, Corentine M. C. Laurin, Rohit Farmer, Stuart J. Conway, Gunnar Boysen, S. Joshua Swamidass, Grover P. Miller

**Affiliations:** 1 Department of Pathology and Immunology, Washington University-St. Louis, St. Louis, MO 63130, USA; noahflynn@wustl.edu (N.R.F.); mickward1029@gmail.com (M.D.W.); rohit.farmer@gmail.com (R.F.); 2 Department of Biochemistry and Molecular Biology, University of Arkansas for Medical Sciences, Little Rock, AR 72205, USA; MADavis@uams.edu; 3 Department of Chemistry, University of Oxford, Oxford OX1 3TA, UK; corentine.laurin@yale.edu (C.M.C.L.); stuart.conway@chem.ox.ac.uk (S.J.C.); 4 Department of Environmental and Occupational Health, University of Arkansas for Medical Sciences, Little Rock, AR 72205, USA; GBoysen@uams.edu

**Keywords:** bromodomain, inhibitor, isoxazole, model, deep neural network, pathway, bioactivation, reactive metabolite, quinone, glutathione

## Abstract

The 3,5-dimethylisoxazole motif has become a useful and popular acetyl-lysine mimic employed in isoxazole-containing bromodomain and extra-terminal (BET) inhibitors but may introduce the potential for bioactivations into toxic reactive metabolites. As a test, we coupled deep neural models for quinone formation, metabolite structures, and biomolecule reactivity to predict bioactivation pathways for 32 BET inhibitors and validate the bioactivation of select inhibitors experimentally. Based on model predictions, inhibitors were more likely to undergo bioactivation than reported non-bioactivated molecules containing isoxazoles. The model outputs varied with substituents indicating the ability to scale their impact on bioactivation. We selected OXFBD02, OXFBD04, and I-BET151 for more in-depth analysis. OXFBD’s bioactivations were evenly split between traditional quinones and novel extended quinone-methides involving the isoxazole yet strongly favored the latter quinones. Subsequent experimental studies confirmed the formation of both types of quinones for OXFBD molecules, yet traditional quinones were the dominant reactive metabolites. Modeled I-BET151 bioactivations led to extended quinone-methides, which were not verified experimentally. The differences in observed and predicted bioactivations reflected the need to improve overall bioactivation scaling. Nevertheless, our coupled modeling approach predicted BET inhibitor bioactivations including novel extended quinone methides, and we experimentally verified those pathways highlighting potential concerns for toxicity in the development of these new drug leads.

## 1. Introduction

Targeted cancer therapeutics boast superior health outcomes relative to traditional chemotherapy drugs; however, hepatotoxicity poses a major clinical concern for patients undergoing treatment with those drugs [1,2,3,4]. A well-established initiating event, especially for idiosyncratic drug-induced liver injury, is the metabolic bioactivation of drugs [5,6,7]. Assessment of the drug bioactivation risk traditionally relies on experimental studies incurring high costs in time, effort, and resources as well as expertise. To mitigate this expense, we have developed metabolism and bioactivation models to increase the efficiency of drug development. First, we designed deep neural models to predict the formation of specific reactive metabolites including epoxides [8] and quinone species [9], and the bioactivation of structural alerts such as furans, phenols, nitroaromatics, and thiophenes [10]. Importantly, the quinone model is capable of predicting the formation of other reactive metabolites within the broader class of conjugated electrophiles, despite its name. Second, we recently reported a novel approach for predicting the structures of metabolites arising from bioactivations [11]. The information could facilitate their experimental identification as well as the construction of possible metabolic pathways. Third, the toxicological relevance of reactive metabolites often depends on the ability to adduct with and modify biological molecules, such as proteins, glutathione, and DNA [12,13,14], and therefore, we developed models to predict adduct formation [9,15]. These computational analyses are more accessible than experiments and provide an opportunity to readily explore potential relationships between molecular structure and bioactivation that lead to testable hypotheses. In practice, we couple high throughput computational studies with experimental efforts to validate predicted relationships and reveal model shortcomings for further refinement into practical tools, as shown through our work on terbinafine [16], thiazoles [17,18], and diphenylamine nonsteroidal anti-inflammatory drugs [19]. In this study, we applied our novel computational and experimental strategy to reveal the potential bioactivation liabilities of bromodomain and extra-terminal (BET) inhibitors.

BET inhibitors comprise a growing class of anti-cancer agents, [20,21] including at least 24 drug candidates in Phase I clinical trials [22,23,24]. These molecules target proteins that possess bromodomains, which are modules that are capable of binding acetylated lysine residues present on histones and many other proteins [25,26]. That action regulates the transcription of oncogenes and anti-apoptotic proteins [22,27]. Consequently, molecules mimicking the modified amino acid are effective at disrupting the bromodomain functions in cancer cells. A number of BET bromodomain inhibitors, including I-BET151, incorporate the 3,5-dimethyl-isoxazole motif as an isostere of acetyl-lysine for its mode of action [28,29,30,31,32,33]. I-BET151 shows efficacious inhibition against leukemic cell line proliferation [33,34] and has progressed to clinical trials [35]. Similar to other early BET inhibitors, I-BET151 induces unwanted toxic side effects that are often attributed to low selectivity amongst different bromodomains and other toxic mechanisms based on elevated liver enzyme and bilirubin levels that are indicative of drug-induced liver injury [36,37]. New generations of BET inhibitors seek to capitalize on the 3,5-dimethylisoxazole motif [28,29,30,31,32,38,39,40,41,42,43,44,45,46] and incorporate more molecular diversity to improve bromodomain (BRD) selectivity, such as between BRD1 and BRD2. This strategy could potentially reduce pan-BET inhibitor-associated side effects yet the risks for other adverse outcomes, such as drug-induced liver injury, may persist. Isoxazole metabolism and bioactivation resulting in toxicity is rare [47] but possible. Isoxazole substituents might potentiate bioactivation risks in which the metabolism generates reactive conjugated electrophilic metabolites, such as cyanoacroleins [48,49] or enimines [50,51], that react with glutathione. To our knowledge, there are no investigations of these types of bioactivations or others for isoxazole-containing BET inhibitors. We hypothesized that coupling our bioactivation, metabolite structure, and reactivity deep neural models would predict bioactivation pathways for the 3,5-dimethyl-isoxazole motif present in BET inhibitors.

Herein, we carried out a novel coupled modeling strategy to predict bioactivation pathways involving isoxazoles and validated them experimentally for selected BET inhibitors that are currently in development. The identification of possible bioactivation pathways for isoxazoles involved coupling deep neural models for quinone species formation, metabolite structures, and then biomolecule reactivity, as described previously [19]. Next, we assessed the capacity of our quinone model to identify potential electrophilic metabolites generated for isoxazole-containing molecules in the Accelrys Metabolite Database (AMD). For select molecules, bioactivation outputs were used to predict the metabolite structures from reactions and then, reactivity with glutathione. As a practical application of this approach, we predicted possible bioactivation pathways for 32 isoxazole-containing BET inhibitors varying in molecular structure and selectivity toward the first bromodomain of bromodomain containing protein 4 (BRD4(1)) [28,52]. Subsequently, we selected the following two promising drug leads: OXBFD02 and OXBFD04. These molecules differ in the substitution of a phenyl group, with pyridine leading to a significantly enhanced half-life and the affinity of BRD4(1) for OXFBD04 relative to OXFBD02 [28]. In this analysis, we also included I-BET151, which is representative of a promising first generation BET inhibitor [34,53], that may undergo bioactivation. We carried out metabolism studies with those molecules using human liver microsomes. Reactions included dansylated glutathione to trap reactive metabolites and track adducts using the fluorescent dansyl tag [19,54,55]. A subsequent mass spectrometric (MS) characterization of the glutathione adducts provided a way to infer structures of the observed reactive metabolites. When combined with the observed reaction metabolites, we were able to construct putative bioactivation pathways for the BET inhibitors and then compare the findings to model predictions.

## 2. Results

### 2.1. Mining the Accerlys Metabolite Database for Possible Isoxazole Bioactivations

Given the limited examples of isoxazole bioactivations in the literature, we mined examples of molecules and reactions present in the AMD for a broader assessment of possible bioactivations. Of the 20,736 parent molecules, we found 344 isoxazole-containing molecules. Of the 344 isoxazole-containing molecules, 12 formed electrophilic conjugated systems via two different reaction mechanisms involving the isoxazole (Figure S1). Using ECFP6-derived fingerprints, the set of 344 isoxazole-containing molecules and the subset of 12 bioactivated molecules each had high internal diversities [56] of 0.85 and 0.70, respectively, indicating the representation of a variety of different molecular structures. The subset of 332 non-bioactivated molecules had a low similarity to the nearest neighbor [56] from the bioactivated subset of 0.212. Two molecules involved the oxidation of the isoxazole fused to another ring, that ultimately led to cleavage of the isoxazole (Figure S1A). The resulting diketo metabolite may be reactive due to conjugation with an adjacent double bond. The inferred metabolite for one molecule is known to be reactive with glutathione, while the metabolites for the other molecule have no evidence of reactivity towards glutathione (Figure S2). The ten other electrophilic conjugated systems involved a 4-amino-5-methyl-isoxazole bioactivated into an enimine (Figure S1B) and all of them were reactive toward glutathione at the methyl group, emphasizing a pattern of bioactivation and reactivity.

### 2.2. Assessment of Model Accuracy to Predict Electrophilic Conjugated Systems Involving Isoxazoles

**Figure 1:**
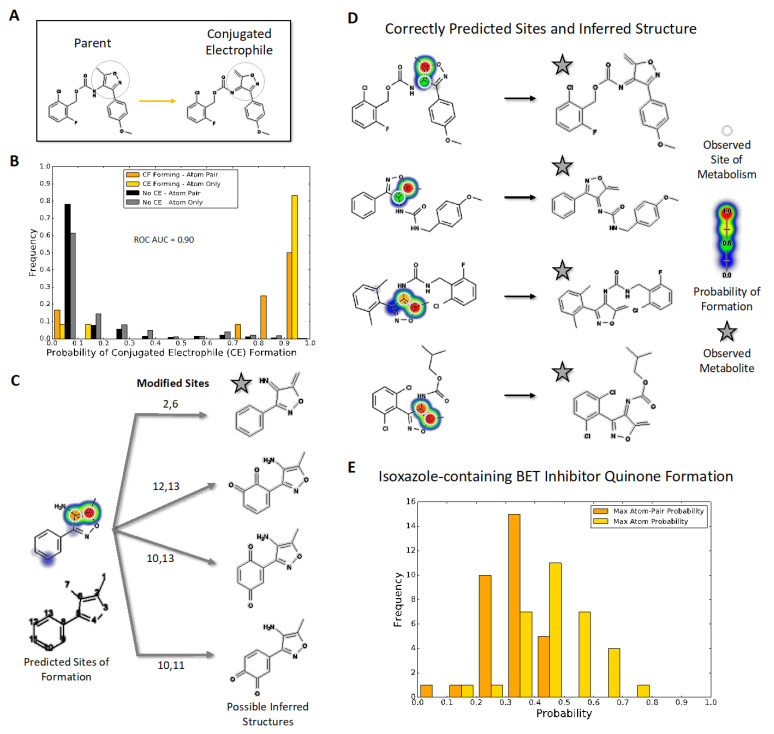
Quinone model predictions for bioactivation of isoxazole-containing molecules (**A**) Compounds with an isoxazole ring, shaded in grey, can be metabolized into reactive electrophiles such as the illustrated enimine. (**B**) The quinone model identifies which isoxazole-containing molecules will undergo bioactivation into a conjugated electrophile involving the isoxazole ring with 90% AUC accuracy. The histogram uses normalized frequencies; therefore, the scale is balanced for the 332 negative (not bioactivated) molecule predictions and the 12 positive (bioactivated) molecule predictions. (**C**) The quinone structure inference model can generate possible quinone and enimine metabolite structures for a queried compound. (**D**) For all 10 enimine-forming molecules, the quinone structure inference model correctly predicts the reported metabolite structure when using the highest atom-pair probability. (**E**) Likelihood of conjugated electrophile formation among drug leads for bromodomains, predicted using the quinone model with two types of assessment (see text).

The identification of bioactivated 4-amino-5-methyl-isoxazoles provided a test set for assessing the ability of coupled models to predict complete bioactivation pathways for enimine formation, metabolite prediction, and biomolecular reactivity. We then assessed model accuracy toward predicting the occurrence and sites of bioactivations for isoxazole-containing molecules within AMD (Figure 1A). First, model predictions identified molecules known to undergo bioactivation with an AUC of 0.90 (0 to 1.0 scale) (Figure 1B) with two exceptions in which bioactivation led to the cleavage of the isoxazole (Figure S2). These findings contrasted with very low predictions for isoxazoles that did not undergo bioactivation, indicating the ability of the model to distinguish between the two possibilities. Second, the model accurately predicted which atoms are the sites of metabolism, leading to the enimine for all ten molecules possessing the 4-amino-5-methyl-isoxazole. Given those positive results, we inferred molecular structures for the corresponding reactive metabolites (Figure 1C). The algorithm predicts many possible metabolite structures; therefore, we scaled their likelihood based on the associated highest atom-pair score from the quinone formation model and reported the most probable one as shown in Figure 1D. This approach led to the correct identification of the experimentally identified reactive metabolites for all ten molecules, whose 4-amino-5-methyl-isoxazole group underwent conversion into an enimine (Figures S3 and S4). Third, we predicted the reactivity of those isoxazole enimines with glutathione. As expected, all ten metabolites were highly reactive with glutathione at the correct site of adduction. Further substituent analysis also revealed that the model predictions align with common knowledge of metabolism and bioactivation, namely the deactivating effects of a phenyl to a pyridine and methyl to ethyl substitutions [57] (Figures S5 and S6). Taken together, the coupling of models provided accurate predictions of 4-amino-5-methyl-isoxazole bioactivations, the resulting metabolites, and subsequent reactivity toward glutathione. 

### 2.3. Application of Coupled Modeling Approach to Predict Bioactivation of BET Inhibitors

Given the importance of BET inhibitors, we used our coupled modeling approach to predict the reactive metabolites and potential for glutathione conjugation for 32 bromodomain inhibitors containing 3,5-dimethyl isoxazoles [28,30,31] (Figures S7 and S8). The quinone model predicted that the inhibitors had an elevated relative risk of 2.7 (*p*-value < 0.001) for undergoing bioactivation relative to the isoxazole-containing molecules from our original validation set that do not undergo those reactions (Figure 1E). However, the inhibitors also had a lower relative risk of 0.45 compared to positive controls (*p*-value < 0.01). Based on the appropriate scaling of quinone model scores to actual probabilities [9], we interpret this trend to mean these molecules are of elevated bioactivation risk and, thus, this outcome prompted our subsequent experimental investigation to validate the predictions. The quinone model predictions also demonstrate general, expected trends for substituent effects promoting quinone formation (Figure S7). Specifically, the modification of the distal phenyl group with methoxy groups decreased bioactivation, while fluorine and chlorine atoms had no effect on the bioactivation potential, regardless of the position on the ring. Lastly, the substitution of that phenyl group with pyridine led to a general decrease in the overall bioactivation predictions.

**Figure 2:**
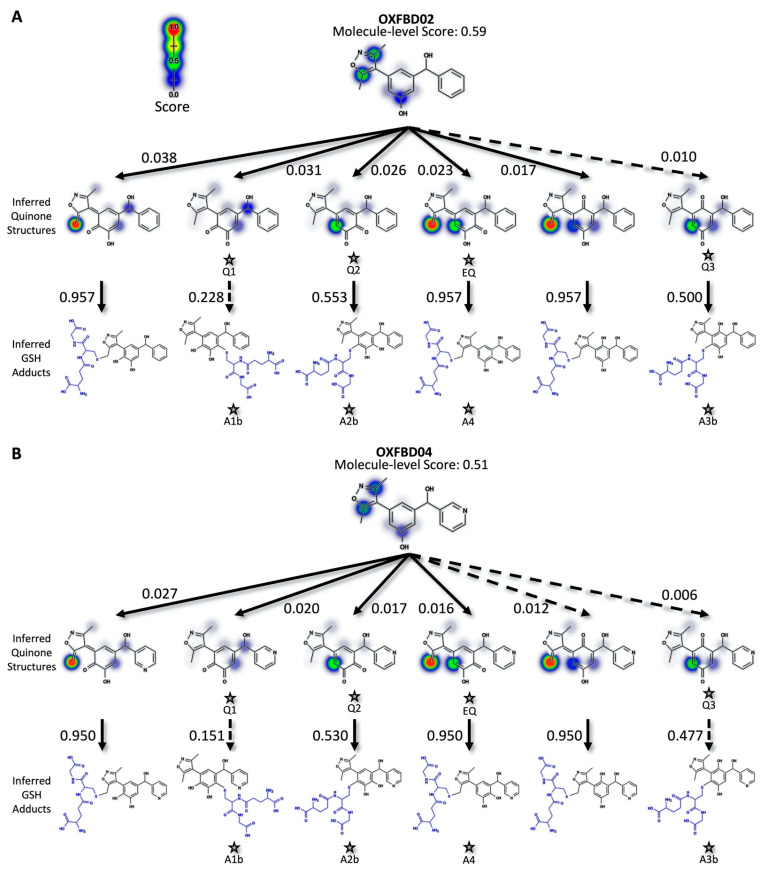
Predicted bioactivation pathways and subsequent adducts for OXFBD molecules. (**A**) Top six model-derived bioactivation pathways for OXFBD02. The top six pathways split evenly between traditional (Q) and extended quinone-methides (EQ) involving the isoxazole and are ranked based on the indicated quinone model score. EQs were highly reactive to glutathione but traditional quinone reactivity was only moderate to low, as reflected in the reactivity prediction values. Dashed arrows denote the transformations for which the predictions are below the model’s binarization threshold. Stars show the predicted glutathione adducted metabolites observed experimentally (Figure 3). (**B**) The top six pathways split evenly between traditional (Q) and extended quinone-methides (EQ) and are ranked based on the indicated quinone model score. EQs were highly reactive to glutathione but traditional quinone reactivity was only moderate to low, as reflected in the reactivity prediction values. Dashed arrows denote the transformations for which the predictions are below the model’s binarization threshold. Stars show the predicted glutathione adducted metabolites observed experimentally (Figure 3).

For more in-depth analyses, we chose three inhibitors for modeling possible metabolic pathways and validating them experimentally. Specifically, we selected an earlier generation BET inhibitor, I-BET 151 [34,53], and in-development drug leads [28], OXFBD02 and OXFBD04, which differ only in the presence of a phenyl substituent versus pyridine, respectively. The quinone model predicted molecule-level bioactivation scores of 0.59 (OXFBD02), 0.51 (OXFBD04), and 0.54 (I-BET151). The substitution between OXFBD02 and OXFBD04 then led to a slight decrease in bioactivation. Application of the structure inference model indicated that all three of the inhibitors underwent bioactivation into a quinone species, including a surprising subset involving the isoxazole ring. These quinone species are novel in comparison to the enimines observed for the AMD set of molecules; however, the formation of those reactive metabolites is not possible for these compounds. To infer and rank the most probable quinone metabolite structures, we used the more reliable atom-pair score for metabolites (Figure 1B) instead of the atom scores, despite the equivalent AUC performance (Figure S9). This performance difference may reflect unlikely outcomes, such as diene formation with both methyl groups when only using atom scores. Furthermore, atom-pair predictions that do not correspond to an inferred structure produced by the structure inference model are discarded and the retained predictions, which map to an inferred quinone structure, are ranked. We predicted glutathione adduct structures for the corresponding quinone species with the highest atom-level reactivity score, and inferred possible bioactivation pathways for OXFBD02, OXFBD04, and I-BET151, as shown in Figure 2 and Figure S10. The number of reported pathways of the most probable quinone metabolites and their most probable adducts was expanded to include all of the experimentally observed metabolites from the parent molecules, with a minimum of the three top pathways if the parent molecule had no experimentally observed metabolites. The highest predictions for bioactivations were relatively low and did not vary significantly when yielding different reactive metabolites. Moreover, the resulting metabolites with conjugated systems possessing a methylene were more reactive with glutathione than quinones in all cases. 

### 2.4. OXFBD02 Underwent Extensive Metabolism and Bioactivation Down Competing Pathways Based on Experimental Studies

**Figure 3:**
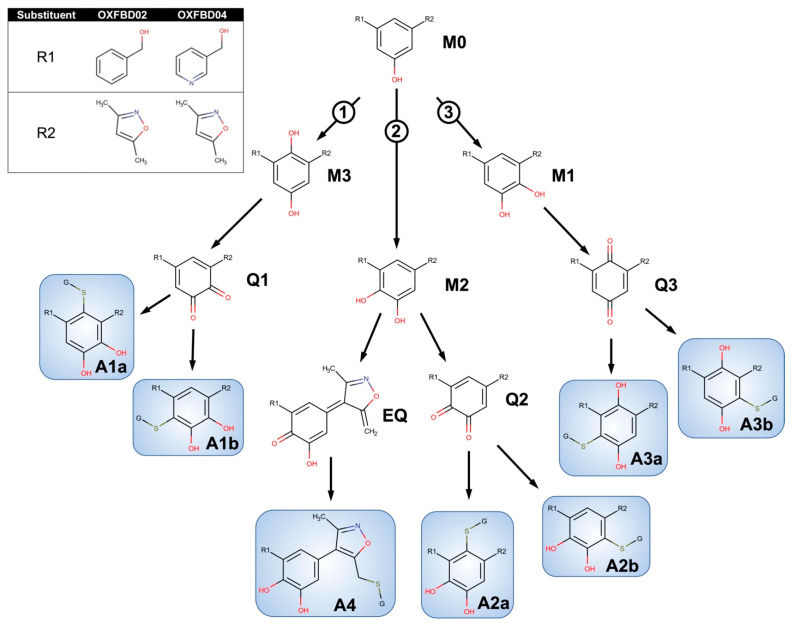
Competing bioactivation pathways for OXFBD series of BET inhibitors. OXFBD02′s and OXFBD04′s bioactivation pathways require an initial hydroxylation of the internal phenyl group. The three potential sites give rise to three competing pathways, as indicated by circled numbers. Pathways 1 and 2 show hydroxylations yielding an *ortho*-hydroquinone. Pathway 1 leads to an *ortho*-quinone and a subsequent pair of glutathione adduct isomers (A1a and A1b). In contrast, Pathway 2 bifurcates, leading to either an *ortho*-quinone and a pair of glutathione adduct isomers (A2a and A2b), or a single glutathione adduct arising from an extended quinone-methide involving the isoxazole (A4). Pathway 3 involves an initial formation of a *para*-hydroquinone followed by a *para*-quinone trapped into a pair of glutathione adduct isomers (A3a and A3b). Analysis of adducts then yields characteristic patterns for pairs of quinones except for the extended quinone in which there is one possibility.

In general, the OXFBD02 bioactivations with microsomal reactions involved sequential oxidations, leading to the formation of quinone species trapped by dansyl glutathione. As shown in Figure 3, an initial hydroxylation of OXFBD02 creates three pathways leading to mono-hydroxylated metabolites (M1, M2, and M3) that can undergo further oxidation into quinone species (Q1, Q2, and Q3) and, in one case, an extended quinone-methide (EQ). Pathways 1 and 2 reflect hydroxylation yielding an *ortho*-hydroquinone. Pathway 1 leads to an *ortho*-quinone and, subsequently, a pair of glutathione adduct isomers (A1a and A1b). In contrast, Pathway 2 bifurcates, leading to either an *ortho*-quinone and a pair of glutathione adduct isomers (A2a and A2b), or a single glutathione adduct arising from an unusual extended quinone-methide (A4). Pathway 3 involves an initial formation of a *para*-hydroquinone followed by a *para*-quinone trapped in a pair of glutathione adduct isomers (A3a and A3b). Taken together, the occurrence of all of the pathways would lead to a 2:2:2:1 pattern of chromatographically resolved adduct peaks.

**Figure 4:**
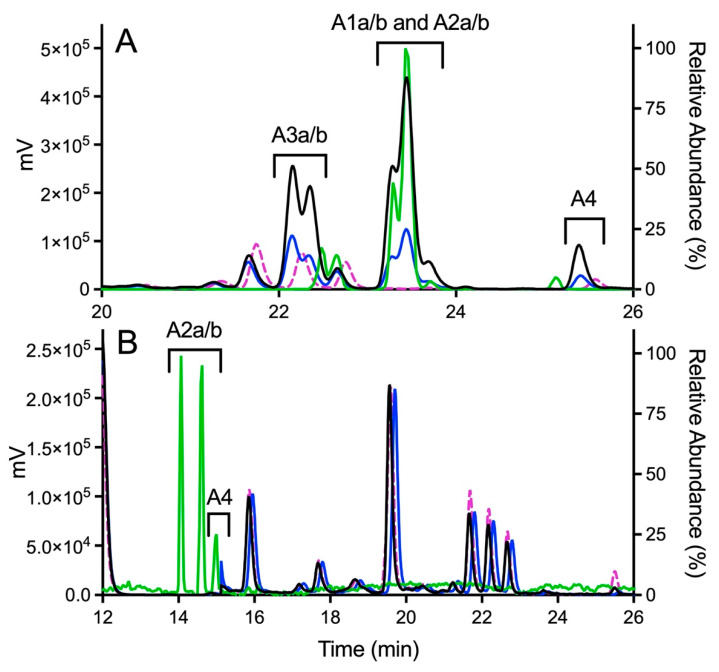
Fluorescence and MS chromatograms of putative quinone adducts for OXFBD02 and OXFBD04 from human liver microsomal reactions. (**A**) OXFBD02 was bioactivated into six fluorescently and mass spectroscopically detectable dansyl-glutathione adducted quinone-species metabolites. (**B**) OXFBD02 was bioactivated into three fluorescently and mass spectrometrically detectable dansyl-glutathione adducted quinone-species metabolites. In both panels, data displayed on the plot are as follows: no metabolic reaction (negative control, pink dotted line), 30 min reaction with fluorescence detection (blue solid line), 60 min reaction with fluorescence detection (black solid line), and 60 min reaction with mass detection (green solid line). The left Y-axis corresponds to data collected from fluorescence detection, whereas the right Y-axis corresponds to data collected from mass detection. Adduct identities are labeled according to the naming strategy displayed in Figure 3 and based on patterns of eluted adducts, as discussed in Results.

Experimental studies revealed an extensive metabolism of OXFBD02 based on chromatographic resolution and the combined detection of analytes by fluorescence and MS. The absence of an NADPH-regenerating system (negative control) indicated many background fluorescence signals from the dansyl glutathione reagent or other reaction constituents (Figure 4A). The complete reaction led to multiple new peaks in a 2:3:1 pattern that increased over time, as expected for metabolites. All of the peaks were presumably dansylated glutathione adducts, given that the parent molecule was spectrally silent under detection conditions. In support of that possibility is the elution of those peaks after the dansyl glutathione trap, as reported for adducts in other studies [19,54,55]. MS analysis of analytes provided further evidence for OXFBD02′s bioactivation into adducted quinone species. The initial total ion scans indicated OXFBD02′s (295 *m*/*z*) oxidation into five mono-hydroxylated metabolites (311 *m*/*z*) and three di-hydroxylated metabolites (327 *m*/*z*) (Figure 3 and Figure S11). These hydroxylated metabolites underwent further oxidation, leading to a reactive quinone species and the extended-quinone methide trapped by dansyl glutathione. 

**Figure 5:**
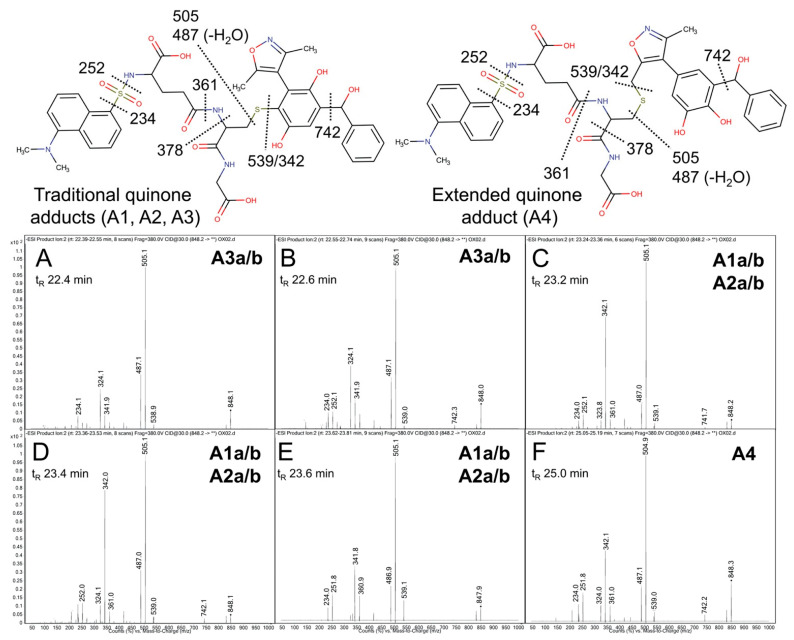
Mass spectrometric product ion spectra for predicted quinone metabolite adducts for OXFBD02. The results show the integrated spectra and product ion fragments for peaks eluting in a 2:3:1 ratio based on intensity in Figure 4A. Characteristic dansyl glutathione fragments *m*/*z* 234, 252, 361, 378, 487, 505, and 539 have been reported previously in negative ion mode by Gan and colleagues [54,55]. The site of hydroxylation did not impact the observed fragment patterns. Assignments for the respective fragments are indicated in the accompanying molecular structures for the corresponding adducts. Peak assignments for adducts (Figure 3) are discussed in detail under Results. (**A**,**B**) shows spectra for the probable pair of adduct isomers (A3a/b) from a traditional quinone in Pathway 3 (Q3). (**C**–**E**) depict the unresolved pairs of adduct isomers from Pathway 1 (A1a/b) and Pathway 2 (A2a/b). Finally, spectra for the last eluting peak (**F**) corresponds to the adduct (A4) from the extended quinone methide (EQ) in Pathway 2.

Product ion mass spectrometry analyses confirmed the expected parent masses and identified the characteristic fragments for the expected adducts. As observed by fluorescence (Figure 4A), scans for the adduct total ion mass of 849 *m*/*z* revealed a chromatographic resolution of three analyte clusters. There was a doublet of peaks followed by a triplet and then a single, late-eluting peak, suggesting the formation of adduct isomers. All of the analytes possessed the 742 *m*/*z* product ion after loss of the characteristic phenoxymethyl group for OXFBD02, indicating that none of the adducts involved the oxidation of that group. Moreover, putative adducts yielded the following signature product ions from the fragmentation of dansyl glutathione: 234, 252, 361, 378, 487, 505, and 539 *m*/*z* (Figure 5) [54,55]. Nevertheless, none of the product ions were distinguishing features among *ortho*- and *para*-quinone isomeric adducts or even that for the extended quinone adduct. We then leveraged the chromatographic 2:3:1 pattern for the eluted adducts to infer their identification (Figure 4A,B). We inferred that the triplet of analytes reflected overlapping elution of the two doublets of *ortho*-quinone adducts (A1a/b and A2a/b) due to significant structural similarities. The larger central peak corresponded to the additivity of fluorescence and mass responses for the co-eluted *ortho*-quinone adducts. The preceding peak doublet reflected the pair of *para*-quinone adducts (A3a/b). Finally, the lone, smaller, late-eluting peak was presumably the single extended quinone-methide adduct. Its elongated structure would interact more strongly with the C18 column than the other adducts, resulting in the longest observed retention time. Definitive evidence for adduct identities would require nuclear magnetic resonance spectroscopy, which is beyond the scope of this study. Nevertheless, the combination of fluorescence and mass responses with the chromatographic pattern of elution indicated that OXBFD02 underwent bioactivation, mainly into reactive *ortho*- and *para*-quinones and, to a lesser extent, an atypical extended quinone-methide.

### 2.5. Metabolism and Bioactivation of OXFBD04 Was Very Limited According to Experimental Studies

**Figure 6:**
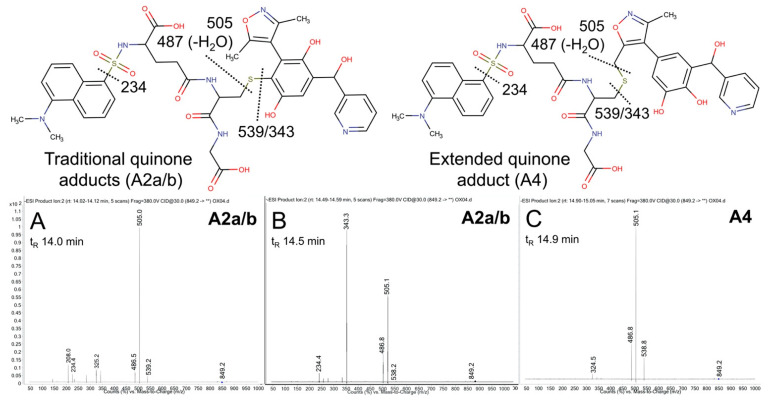
Mass spectrometric product ion spectra for predicted quinone metabolite adducts for OXFBD04. The results show the integrated spectra and product ion fragments for peaks eluting in a 2:1 ratio based on intensity in Figure 4. Characteristic dansyl glutathione fragments *m*/*z* 234, 252, 361, 378, 487, 505, and 539 have been reported previously in negative ion mode by Gan and colleagues [54,55]. The site of hydroxylation did not impact the observed fragment patterns. Assignments for the respective fragments are indicated in the accompanying molecular structures for the corresponding adducts. Peak assignments for adducts (Figure 3) are discussed in detail under Results. (**A**,**B**) shows spectra for the probable pair of adduct isomers (A2a/b) from a traditional quinone (Q2), while (**C**) shows that for the lone, lower intensity peak for A4 arising from the extended quinone methide (EQ).

The possible metabolic pathways for OXFBD04 are superficially the same as those for OXFBD02 based on their structural similarities (Figure 3). Nevertheless, substitution of a phenyl group with pyridine led to significantly different extents of metabolism and bioactivation. The fluorescence chromatogram from the human liver microsomal reactions indicated no distinguishing peaks from the background (Figure 4B) yet labeled adducts could be beyond the limits of detection and/or co-eluting with background peaks. Complementary total ion scans showed that OXFBD04 (296 *m*/*z*) underwent metabolism into only two mono-hydroxylated metabolites (312 *m*/*z*) (Figure S12). Initial scans of the proposed quinone species and extended quinone-methide adduct, with total ion masses at 850 *m*/*z*, yielded a 2:1 pattern with two peaks of similar intensity followed by a smaller third one (Figure 4B). Product ion mass spectrometry resulted in several characteristic dansyl glutathione fragments (324, 487, 505, and 539 *m*/*z*) for putative trapped quinone species adducts (Figure 6), albeit with less abundant responses than those observed for the OXFBD02 adducts. As with OXFBD02, no product ions were distinguishing features among the possible adducts. Nevertheless, the elution pattern was consistent with a pair of quinone adduct isomers followed by the extended quinone-methide adduct. The identity of the isomeric adducts are likely to be A2a/b due to their origination from the same mono-hydroxylated metabolite (M2). In following, OXFBD04 seems to preferentially undergo metabolism and subsequent bioactivation down a single pathway among three possibilities.

### 2.6. There Was No Experimental Evidence of I-BET151 Bioactivation Despite Limited Metabolism

Analysis of human liver microsomal reactions for I-BET151 indicated limited metabolism. As observed for OXFBD04, there were no apparent unique fluorescent peaks generated during the metabolism of I-BET151 as a function of time (Figure S13). The subsequent total ion scans showed elution of the parent drug (417 *m*/*z*) and two mono-hydroxylated metabolites (433 *m*/*z*) (Figure S14), but no observable di-hydroxylated or tri-hydroxylated metabolites. Moreover, total ion mass spectrometry revealed no peaks corresponding to predicted parent masses for 952 or 984 *m*/*z* corresponding to possible glutathione-reactive quinone species metabolites from I-BET151 metabolism. Taken together, MS and fluorescence analyses failed to reveal any evidence for a quinone species adduct, suggesting that I-BET151 does not undergo bioactivation.

## 3. Discussion

### 3.1. Quinone Model Predictions for Reactive Non-Quinones Involving 4-Amino-5-Methyl-Isoxazole

Despite its name, our quinone model was able to predict the bioactivation of molecules containing 4-amino-5-methyl-isoxazole into rare, and yet observed, enimines [50,51]. This new finding suggests that our model has utility in identifying undiscovered metabolites and the impact of substituents on those outcomes. Those results did not reflect model bias from the training set. The high diversity in structure within and between test and training sets made that possibility unlikely. In fact, diversity in the training data has taught the model to adjust the magnitude of predictions due to alkyl substituent effects. The replacement of the methyl with an ethyl group on the isoxazole decreased the model scores. This effect reflects the hindrance of the orbitals of the ethyl group to adopt the ethene structure *versus* methylene for the methyl group, as observed in the reported isoxazole enimines. Moreover, the model accurately predicted a decrease in the bioactivation potential between phenyl and pyridine to form quinones when attached to the isoxazole. The trend might reflect the impact of the electron withdrawing nature of the pyridine nitrogen, which suppresses oxidation typically by cytochromes P450 [57,58,59]. After the bioactivation of the 4-amino-5-methyl isoxazole, the exposed methylene was very reactive and was targeted for adduction by glutathione, as reported in the literature [50,51]. These data demonstrated the potential for the quinone model to predict unexpected reactive metabolites other than the traditional quinones within the broad class of conjugated electrophiles and, thus, could have more applications in predicting bioactivations. 

### 3.2. Modeled Bioactivations of the 3,5-Dimethyl Isoxazole Present in BET Inhibitors

As a practical application, we predicted the bioactivation of a series of 32 established and under-development BET inhibitors to determine whether 3,5-dimethyl isoxazole was able to form a reactive metabolite. The isoxazole lacks the 4-amino group, which played a critical role in the previously reported isoxazole bioactivations [50,51]. For this test set, all of the inhibitors displayed a decreased bioactivation risk relative to the molecules known to form enimines. This finding suggests that the 4-amino group on the isoxazole favored bioactivation according to the model. While 3,5-dimethyl isoxazole led to a lower bioactivation potential, the modeled likelihoods were greater than that for isoxazole-containing molecules contained within AMD that do not undergo bioactivation. The model scores varied with substituents on the distal phenyl ring indicating the ability of the model to scale their impact on bioactivation. Specifically, methoxy groups decreased bioactivation, while fluorine and chlorine atoms had no effect regardless of their position on the ring. In addition, the substitution of that phenyl group with pyridine led to a general decrease in the overall bioactivation predictions. These differences may reflect effects or a lack of effects on chemical reactions as well as enzyme specificities but require further study to validate the trends and determine the underlying causes. Consequently, we selected three of these BET inhibitors, namely, OXFBD02, OXFBD04 (in-development drug leads) [28], and I-BET151 (first generation BET inhibitor) [34], for more in-depth modeling analyses and, importantly, confirming the predicted bioactivations with experimental studies. 

### 3.3. Metabolism and Bioactivation of OXFBD02 and 04, but Not I-BET151, into Quinones

The molecular structures of the selected inhibitors impacted the bioactivation possibilities and subsequent interpretation of the findings. OXFBD02 and 04 share a common isoxazole-phenyl scaffold and differ only in the presence of an additional distal phenyl or pyridine substituent, respectively. For the OXFBD molecules, the structural difference led to a 10-fold increase in metabolite stability for OXFBD04 over OXFBD02 in a half-life assay [28]. Our metabolic studies corroborated the observed effect on metabolism and demonstrated the likely dominance of cytochromes P450 in metabolisms that are based on a requirement for NADPH [60]. Surprisingly, the suppressed bioactivation was not due to the deactivating effects of nitrogen in the ring [57,58,59], because the distal pyridine and phenyl substituents did not undergo metabolic activation into reactive quinones (Figure 3). The observed effect has to be indirect, such as modulations of enzyme specificity possibly resulting from the pyridine ring being charged at a physiological pH. Alternatively, the binding mode makes the molecule act more as an inhibitor than a substrate. For the pair of OXFBD molecules, the overall model scores reflected a decrease in the metabolic bioactivation of OXFBD04 over OXFBD02. This finding aligned with the phenyl to pyridine substituent effects on quinone bioactivation observed with the molecules present in AMD. In other words, the quinone model successfully applied the lessons learned from molecules very different from these BET inhibitors, even if the mechanism for this effect remains unclear. I-BET-151 also possesses a distal pyridine substituent, but the scaffold is very different, with the isoxazole linked to a tricyclic moiety making direct comparisons with the OXFBD molecules impossible. 

Metabolism yielded two general possible outcomes for bioactivations; molecules underwent oxidation into traditional *ortho*- or *para*-quinones and novel extended quinone-methides involving the isoxazole. For the top six predictions, modeled OXFBD bioactivations involved multi-step reactions resulting in an even split between both types of quinones and their glutathione adducts. Among them, the model predictions highly favored formation of extended quinone-methides. Overall, four of the six predicted adducts were verified by experimental studies. Nevertheless, experimental studies showed that the dominant OXFBD bioactivations were a formation of traditional quinones with extended quinone-methides being minor metabolites. Moreover, the efforts provided more insights and evidence for reaction steps and specific pathways. The extensively metabolized OXFBD02 yielded mono- and dihydroxylated metabolites that ultimately fed into three competing bioactivation pathways (Figure 3). These possibilities arose from hydroxylation of the phenyl scaffold at one of three sites. Further oxidation yielded a reactive quinone species with two sites of attack by glutathione, so that the subsequent glutathione trapping led to pairs of adduct isomers. Importantly, hydroxylation of the phenyl ring *para* to the isoxazole created an alternate bioactivation pathway leading to an extended quinone-methide involving both isoxazole and phenyl rings. In contrast, the suppressed metabolism of OXFBD04 yielded just two mono-hydroxylated metabolites and corresponding reactive quinones in Pathway 2 (Figure 3). The metabolites of other pathways for OXFBD04 were beyond the limit of detection for our highly sensitive assay, or simply not present. For I-BET151, modeled bioactivations were similar in magnitude as those for OXFBD molecules, yet the pathways led only to extended quinone-methides with a highly reactive methylene group. Unlike those predictions, experimental studies showed minimal I-BET151 metabolism leading to some hydroxylated metabolites and no evidence of any trappable reactive metabolites. Taken together, modeling was capable of predicting a variety of quinones including novel extended quinone-methides but fell short of accurately scaling the likelihood of their formations. Nevertheless, we report the first experimental evidence for novel extended quinone-methides involving the 3,5-dimethylisoxazole scaffold. 

### 3.4. Novelty and Relevance of Extended Quinone-Methides 

Energetically, traditional quinones would be more accessible to oxidative transformations. Those reactions involve breakage of the aromaticity of the phenyl ring only as opposed to the phenyl and isoxazole rings for the extended quinone-methide. In fact, experimental evidence for an extended quinone species is rare [61]. The most well-known examples include the selective estrogen receptor modulators (SERMs) raloxifene and arzoxifene, in which bioactivation of the benzothiophene scaffold results in extended quinone species with very short half-lives and, possibly, minimal toxicity [62,63,64,65,66]. In contrast, bioactivation of the estrogen diethylstilbestrol into an extended quinone species may contribute to the teratogenic effects of the drug, leading to its withdrawal from the market in 1971 [67,68]. It is possible that extended quinone species are more prevalent but escape detection due to their short half-lives and/or insufficient experimental designs to capture their formation. Additionally, there is no uniform structure for an extended quinone species, making detection more challenging. In our case, the quinone model predicted that all three BET inhibitors underwent bioactivation into an extended quinone species and we experimentally validated their occurrence for the two OXFBD molecules. Those findings suggest that formation of the extended quinone-methide may be inherent with the drug scaffold, albeit a very minor pathway. Nevertheless, if the model scores correlate with toxicity, then these results may have implications for these drug leads. The modeled bioactivation scores (0.51 and 0.59) were similar in magnitude to that for estradiol (0.60) from a previous study by our group [9]. Estradiol is well-known to undergo bioactivation and induce toxic risks, suggesting those possibilities may exist for OXFBD molecules [69]. 

### 3.5. Advances in Isoxazole Bioactivation Studies Revealed Current Limitations of Those Efforts

These modeling and experimental studies provided an uncommon, concerted effort to study isoxazole bioactivation, but indicated that gaps remain. While very rapid and accessible, modeling did not accurately scale the relative probabilities of traditional quinones and extended quinone-methides based on model scores. The molecule-level model output is well-calibrated, but atom-pair-level scores can lead to underconfident predictions and are not probabilistic. We partially resolved this problem by computing an optimal threshold for binarizing scores. In future applications, it may be advantageous to interpret scores as probabilistic for assessing confidence in the predictions. Improving the calibration of pair-level model predictions may also improve the rankings of inferred metabolites to better reflect experimental results. Another non-intuitive feature of the quinone model is that atom and atom-pair scores can be very different. Sometimes high predictions do not correspond to valid reactive metabolite structures, with atom scores having less reliability than atom-pair scores. The validity of predictions was improved by using the structure inference model to filter out highly predicted sites that do not produce an inferred structure. For the experimental studies, analysis of the reactive metabolites relied on set reaction conditions and no steady-state kinetics that could be used to extrapolate the potential in vivo relevance of bioactivation pathways [16,18]. Moreover, there are no reported studies investigating and confirming that the bioactivation of isoxazole-containing molecules contributes to in vitro or in vivo toxicity. Nevertheless, knowledge of the relative significance of bioactivation pathways could aid in developing future studies to resolve those issues. 

## 4. Materials and Methods

### 4.1. Materials

Chemical solvents were purchased from Thermo Fisher Scientific (Waltham, MA, USA). Substrate I-BET151 was purchased from Cayman Chemical (Ann Arbor, MI, USA) and substrates OXFBD02 and OXFBD04 were synthesized as reported previously [28,31]. Trapping agent dansyl glutathione trifluoroacetic acid salt was purchased from Toronto Research Chemicals (Toronto, ON, Canada) while internal standard dansylamide was obtained from Millipore Sigma. Reducing agent tris(2-carboxyethyl)phosphine hydrochloride (TCEP) was purchased from Millipore Sigma. Human liver microsomes pooled from 150 donors (HLM150) were purchased from Corning Gentest (Woburn, MA, USA).

### 4.2. Data to Assess Quinone Model Performance toward Isoxazole-Containing Molecules

We identified isoxazole-containing molecules and those undergoing bioactivation from the literature-derived Accelrys Metabolite Database (AMD) to evaluate model performance. We collected reaction data for all of the isoxazole-containing small molecules catalyzed within the human liver using SMARTS patterns for the isoxazole ring. We built a graph database with Neo4j to connect molecules with observed direct and downstream metabolites up to three metabolic steps away. We queried the graph database to find conjugated electrophile species involving an isoxazole ring by applying our previously developed structure inference model, the Metabolic Forest [11], to identify metabolite pairs formed between the isoxazole-containing molecule and direct or downstream metabolites. For identified molecule pairs, the upstream isoxazole-containing molecule was annotated as forming a conjugated electrophile. Then, we queried our graph to determine whether the isoxazole molecules that form conjugated electrophiles had been experimentally observed to react with glutathione. We identified 344 isoxazole-containing molecules in AMD. Of those, 12 molecules formed enimines, and all but one was reactive to glutathione. Some model evaluation molecules (validation set) were also in model training sets, which may bias predictions, and therefore we used a hold-out prediction to ensure that we were evaluating model ability to learn the general principles of the task, rather than memorize the training data. 

### 4.3. Modeling Bioactivation Pathways for Isoxazole-Containing Molecules

We predicted the complete bioactivation pathways for isoxazole-containing molecules by coupling models for quinone (conjugated electrophile) formation, metabolite prediction, and molecular reactivity. First, we revealed the bioactivation sites by modeling the formation of conjugated electrophiles, namely quinones and enimines [9]. This deep neural network model predicts one- and two-step conjugated electrophile formation by identifying atom pairs at which metabolic oxidation may occur to form conjugated electrophile metabolites with an accuracy of 88.2% based on a ROC analysis. In this case, we evaluated the model ability to discriminate between the bioactivated (positives) and non-bioactivated (negatives) isoxazole-containing molecules. The assessments relied on the 12 isoxazole-containing molecules undergoing bioactivation (positives) and 332 non-bioactivated isoxazole-containing molecules (negatives) obtained from AMD. From those results, we calculated model accuracy with a ROC-AUC score based on (1) comparing highest bioactivation scores between positives and negatives and (2) proper assignment of sites for conjugated electrophile formation as recorded in AMD. Second, we used the quinone model outputs to predict structures for the reactive metabolites using the XenoNet model and scaled their likelihood based on the quinone model predictions [70]. This model uses the input molecule to enumerate pathways of metabolite structures while computing likelihood scores for each pathway. Third, we modeled the reactivity of individual quinone or enimine metabolites toward glutathione as a trap [10,15]. Our reaction models scale scores differently, and thus we relied on the quinone model values as the final arbiter of the possible bioactivation potential for isoxazole-containing molecules. We calculated the accuracy of the reactivity model based on the ROC-AUC score using the highest atom score for each molecule and proper identification of the atom reactive toward glutathione, as recorded in AMD. Lastly, we used the Metabolic Forest model to predict glutathione adduct structures based on the glutathione rule within its conjugation rule set [11].

### 4.4. Experimental Bioactivation of Isoxazole-Containing BET Inhibitors as Test Cases

We validated model predictions for the bioactivation of selected BET inhibitors through experimental approaches. All of the reactions employed metabolism by pooled human liver microsomes as a model for the average adult human liver. Wells contained 1 mg/mL HLM150, 1 mM dansyl glutathione, and 50 µM substrate in a 100 mM potassium phosphate buffer with a pH of 7.4 with 0.1% DMSO (co-solvent) and were preincubated for 5 min at 37 °C with shaking at 350 rpm using a BMG Labtech THERMOstar incubator (Ortenberg, Germany). Reactions were then initiated upon addition of a NADPH regenerating system (0.4 μU μL-1 glucose-6-phosphate dehydrogenase, 10 mM glucose 6-phosphate, 2 mM MgCl_2_, 500 μM NADP^+^). Identical mixtures without an addition of the NADPH regenerating system were incubated as negative controls. After 30, 60, or 90 min, aliquots were quenched by adding an 2-fold volume of ice-cold methanol containing an internal standard and a reducing agent (10 μM dansylamide and 5 mM TCEP, respectively) and incubated on ice for 10 min to optimize the precipitation of proteins and a phosphate buffer [71]. After a 2800× *g* centrifugation at 4 °C for 15 min using a Thermo Scientific Sorvall ST 16R Centrifuge (Waltham, MA, USA), the supernatant was transferred to a 96-well full-volume microplate and evaporated to dryness using an Organomation Associates Microvap Nitrogen Evaporator System (Berlin, MA, USA). Dried wells were then resuspended in mobile phase (20:80 H_2_O:CH_3_CN + 0.1% formic acid) for HPLC UV/Vis/Fluorescence and mass spectroscopic analysis, as described in the following section. Each reaction set was performed in triplicate and replicated two to five times.

### 4.5. Characterization of BET Inhibitor Reactive Metabolites Trapped as Glutathione Adducts 

Reactions were analyzed using LC-fluorescence to infer the yields and LC-MS to characterize the structures, as reported previously [19]. Specifically, reaction metabolites were separated using a 4.6 × 150 mm Waters XSelect HSS C18 3.5 µm column heated to 40 °C with a Shimadzu LC-20AB Prominence liquid chromatograph and detected using a Shimadzu RF-10AXL fluorescence detector. Mobile phase consisted of Solvents A (0.1% formic acid/90:10 H_2_O:CH_3_CN) and B (0.1% formic acid/CH_3_CN). A gradient method started with 89% of A and held for 3 min, decreased to 55% of A over 20 min, decreased to 11% of A over 10 min. Solvent A was then increased back to 89% over 2 min and held for the remainder of the run. Flow rate was 1 mL/min and run time was 38 min. The fluorescence detector was set to excitation at 340 nm and emission at 525 nm [54,55,72] to detect the dansyl tag. At 15 min, both gain and sensitivity were increased to improve the visibility of potentially low-yield metabolites. Analyte responses were normalized to the internal standard dansylamide. We then analyzed the metabolites and adducts using mass spectrometry to determine the parent masses and fragmentation patterns to validate the purported structures. Samples were injected onto an Agilent Technologic 1290 Infinity HPLC using the same chromatographic method and column as described above. Analytes were scanned with the Agilent Technologic 6490 Triple Quad LC/MS. The ESI source was operated in negative and positive ion mode, and ion spectra were acquired in full scan mode monitoring the *m*/*z* range of 100–1200 amu. Subsequently, product ion spectra were generated from precursor ions *with* monitoring for fragmentation by collision-induced dissociation with a collision energy of 30 eV and a range of 45–1000 amu in negative ion mode.

## 5. Conclusions

Taken together, our computational and experimental studies yielded the first bioactivation evidence for 3,5-dimethylisoxazole-containing BET inhibitors. In the process, we demonstrated that the deep neural quinone model prediction of enimines indicates the ability to model multiple types of conjugated electrophiles and thus, have broader applications. Improvements in model scaling events relative to one another would further expand on those possibilities. The predicted bioactivation of other novel structural motifs would flag drug scaffolds early in the drug discovery stage to validate concerns before committing significant time and resources in their development. For the BET inhibitors, the occurrence of bioactivations shows a need for subsequent studies to establish relevance in toxicological mechanisms. The findings in this study provide guidance on the potential molecular initiating events to establish the relationship between isoxazole substituents, and their relative bioactivation and subsequent toxic risks. That knowledge can then guide the selection of structural variants with a safer health outcome profile during drug development. Importantly, our combined investigative approach provides a method to not only identify these risks but also facilitate the development of BET inhibitor leads that are less prone to bioactivations.

## Data Availability

The data presented in this study are available in article and Supplementary Materials.

## Supplementary Materials

The following are available online at https://www.mdpi.com/article/10.3390/metabo11060390/s1, Figure S1: Alternate pathways for isoxazole bioactivation into electrophilic conjugated systems, Figure S2: Two examples of AMD pathways involving isoxazole cleavage, Figure S3: Model predicted bioactivation of 4-amino-5-methyl-isoxazole group into enimines, Figure S4: Similarity analysis of isoxazole-containing molecules against the model training set, Figure S5: Model bioactivation predictions for isoxazoles with either a phenyl or pyridine substituent, Figure S6: Model bioactivation predictions for isoxazoles with either a methyl or ethyl substituent, Figure S7: Modeled bioactivation of BET inhibitor drug leads, Figure S8: Model accuracy analysis for molecule- and pair-level scores for predicted conjugated electrophiles, Figure S9: Quinone model atom pair and atom scores are different, Figure S10: Modeled I-BET151 bioactivation pathways, Figure S11: Mass spectroscopic analysis of OXFBD02 microsomal reactions, Figure S12: Mass spectroscopic analysis of OXFBD04 microsomal reactions, Figure S13: Fluorescence and MS chromatograms of putative quinone adducts for I-BET151 from human liver microsomal reactions, Figure S14: Mass spectroscopic analysis of I-BET151 microsomal reactions.

## Abbreviations

The following abbreviations were used in the manuscript: AMD, Accelerys Metabolite Database; AUC, Area Under the Curve; BET, bromodomain and extra-terminal; EQ, extended quinone; LC, liquid chromatography; MS, mass spectrometry; NADPH, nicotinamide adenine dinucleotide phosphate (reduced); Q, quinone; ROC, Receiver Operator Characteristic; TCEP, tris(2-carboxyethyl)phosphine hydrochloride.
