# Supplementary material for: Bioactivation of Isoxazole-Containing Bromodomain and Extra-Terminal Domain (BET) Inhibitors
Source: Metabolites. 2021 Jun 15;11(6):390. doi: 10.3390/metabo11060390 (PMC8232216; doi:10.3390/metabo11060390)
Supplement: Supplementary file 1 [file metabolites-11-00390-s001.zip › metabolites-1232948-supplementary.pdf]

Supporting Information

Bioactivation of isoxazole-containing bromodomain and extra terminal domain (BET) inhibitors

Noah R. Flynn, Michael D. Ward, Mary A. Schleiff, Corentine M. C. Laurin, Stuart J. Conway, Gunnar Boysen, S. Joshua Swamidass and Grover P. Miller

Contents

Fig. S1. Alternate pathways for isoxazole bioactivation into electrophilic conjugated systems.
Fig. S2. Two examples of AMD pathways involving isoxazole cleavage.
Fig. S3. Model predicted bioactivation of 4-amino-5-methyl-isoxazole group into enimines.
Fig. S4. Similarity analysis of isoxazole-containing molecules against the model training set.
Fig. S5. Model bioactivation predictions for isoxazoles with either a phenyl or pyridine substituent.
Fig. S6. Model bioactivation predictions for isoxazoles with either a methyl or ethyl substituent.
Fig. S7. Modeled bioactivation of BET inhibitor drug leads.
Fig. S8. Model accuracy analysis for molecule- and pair-level scores for predicted conjugated electrophiles.
Fig. S9. Quinone model atom pair and atom scores are different.
Fig. S10. Modeled I-BET151 bioactivation pathways.
Fig. S11. Mass spectroscopic analysis of OXFBD02 microsomal reactions.
Fig. S12. Mass spectroscopic analysis of OXFBD04 microsomal reactions.
Fig. S13. Fluorescence and MS chromatograms of putative quinone adducts for I-BET151 from human liver microsomal reactions.
Fig. S14. Mass spectroscopic analysis of I-BET151 microsomal reactions.

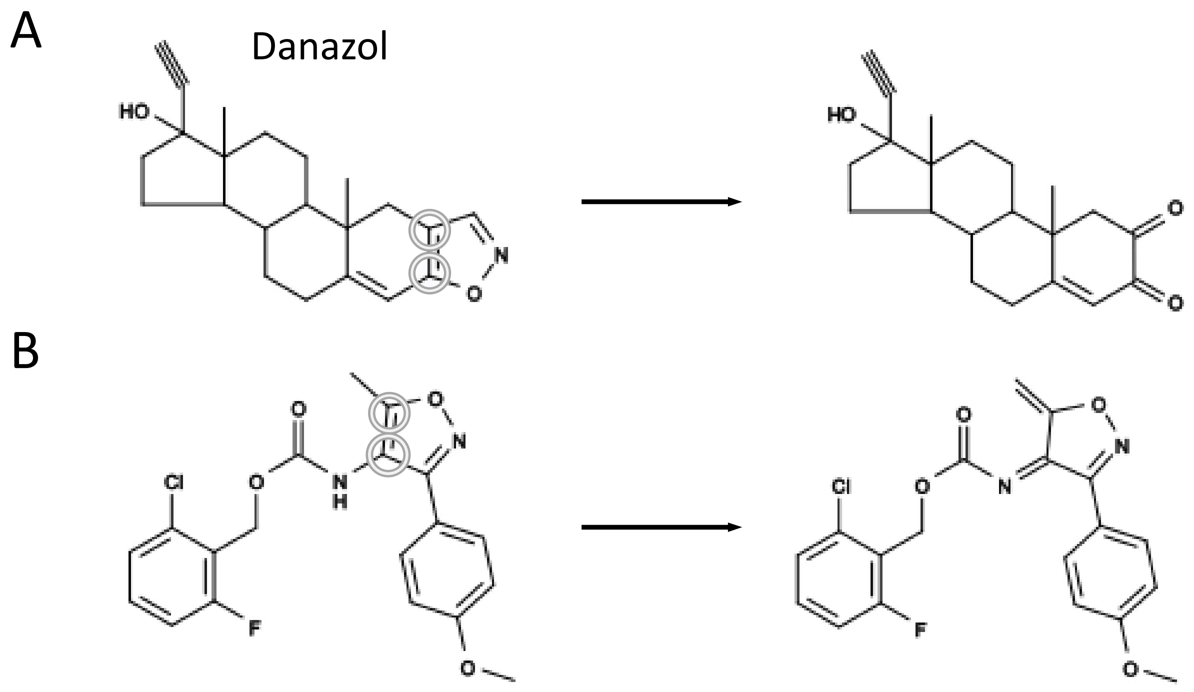

Fig. S1. Alternate pathways for isoxazole bioactivation into electrophilic conjugated systems. **[A]** When fused to a ring, isoxazole metabolism may lead to ring cleavage to yield a diketo metabolite. **[B]** Methyl substitution of the isoxazole makes possible the formation of an electrophilic conjugated system.

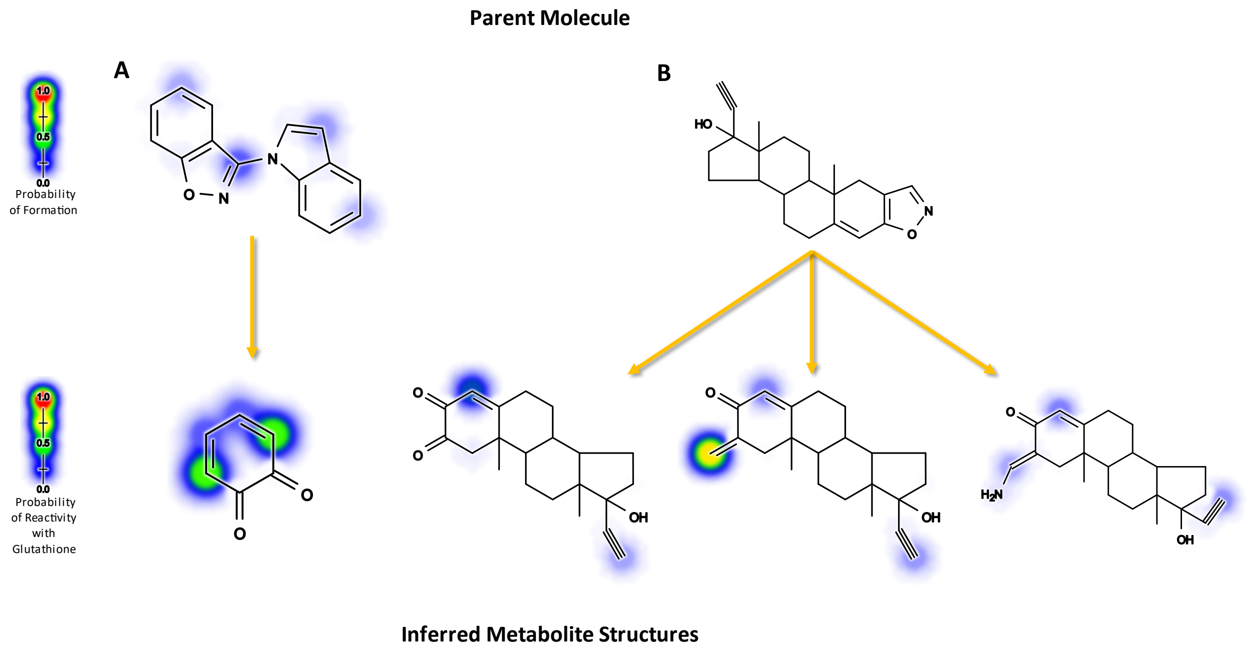

Fig. S2. **Two examples of AMD pathways involving isoxazole cleavage. [A]** The inferred metabolite for one molecule is known to be reactive with glutathione, while **[B]** the metabolites for the other molecule have no evidence of reactivity towards glutathione. For both **[A]** and **[B]**, the model predicted lower formation scores relative to the other isoxazole-containing molecules known to form conjugated electrophiles. In practice, the model may be prone to missing potential bioactivation pathways that require cleavage of the isoxazole.

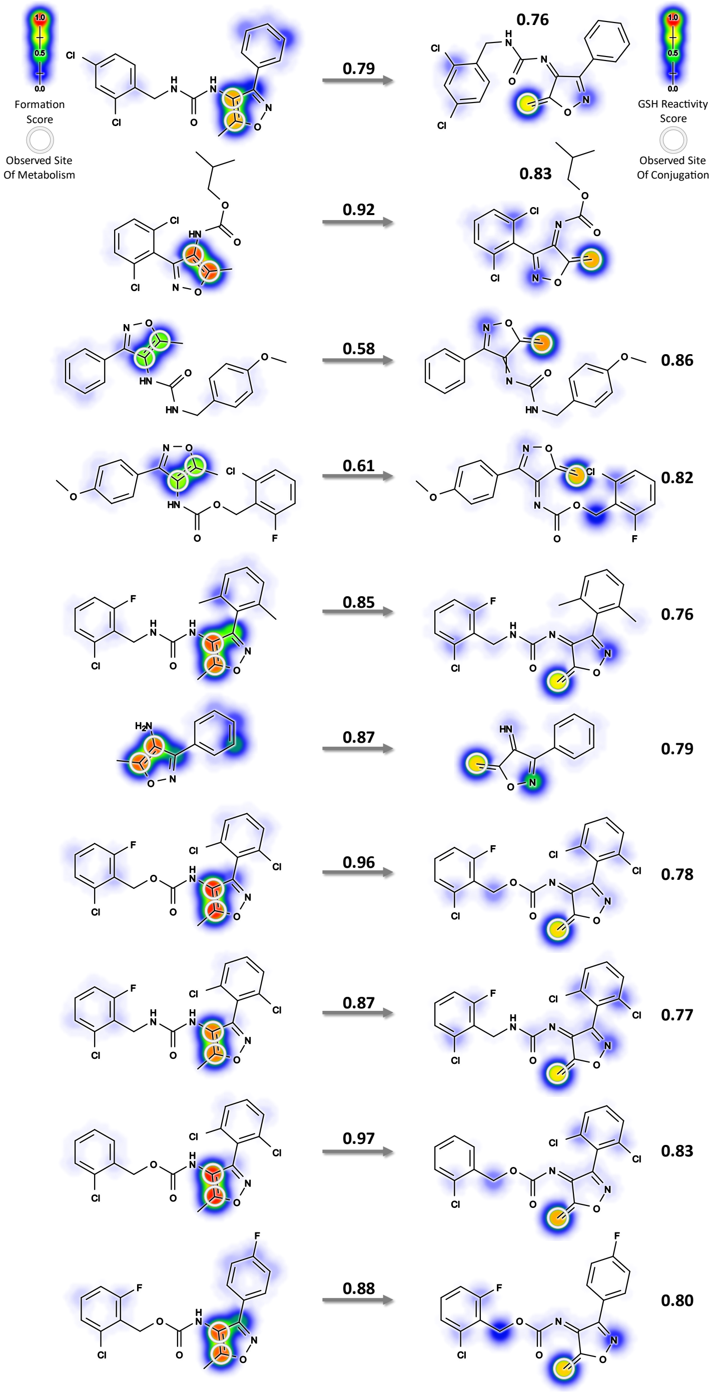

Fig. S3. **Model predicted bioactivation of 4-amino-5-methyl-isoxazole group into enimines.** The left side displays the ten molecules with an overlay of the quinone formation model predictions and numbered arrows that represent max pair-level predictions. The right-hand side displays the inferred metabolite structures with an overlay of the reactivity model predictions for GSH and max site-level reactivity prediction.

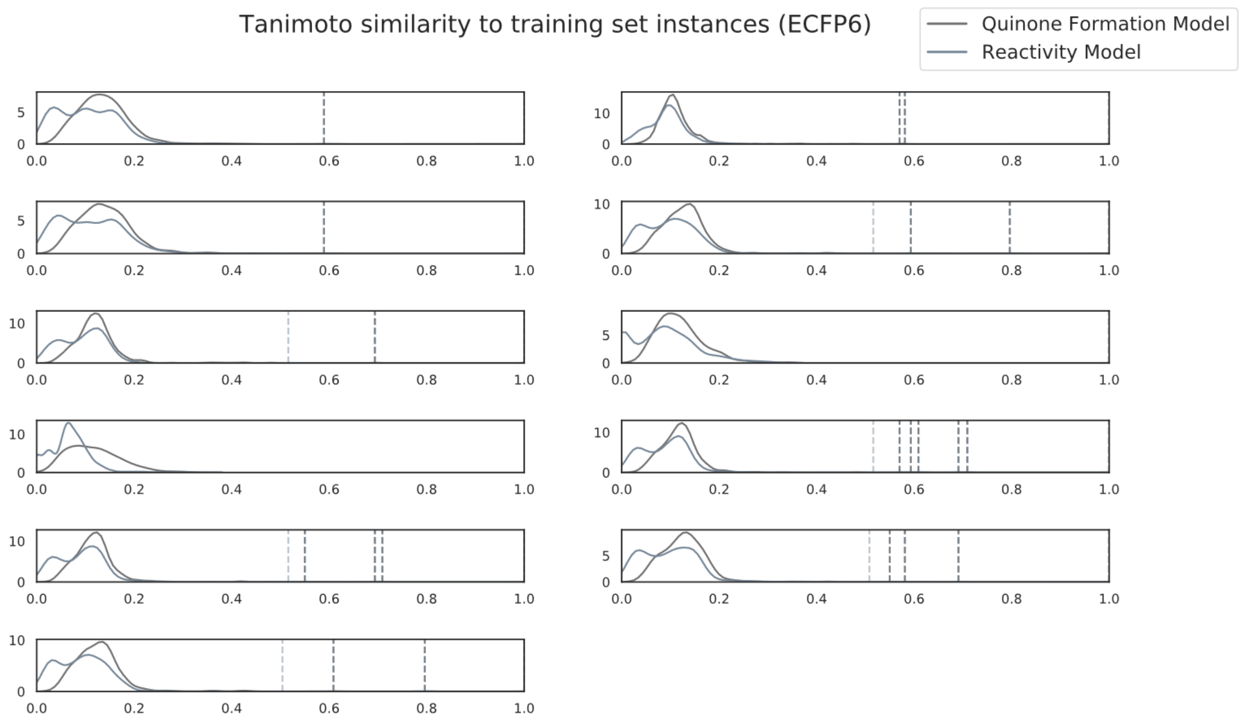

Fig. S4. **Similarity analysis of isoxazole-containing molecules against the model training set.** To contextualize model predictions on the 11 positive isoxazole-containing molecules, we evaluated their Tanimoto similarity and their inferred reactive metabolite's Tanimoto similarity against the quinone model training data set and the reactivity model training set, respectively, on ECFP6-derived fingerprints. The vertical dashed-lines mark Tanimoto similarities above 0.5 for each distribution (darker lines for the quinone model training set and lighter lines for the reactivity model training set). With respect to 718 instances used to train the quinone species formation model, 2, 3, 3, 2 and 1 of the molecules had a Tanimoto similarity greater than 0.5 with 0, 1, 2, 3 and 5 of the training instances, respectively. With respect to 2,803 instances used to train the molecular reactivity model, 5 and 6 of the reactive metabolites had a Tanimoto similarity greater than 0.5 with 0 and 1 of the training instances, respectively. For both models, there is not a high proportion of similar training set instances to the molecules and reactive metabolites being validated to affect the assessment.

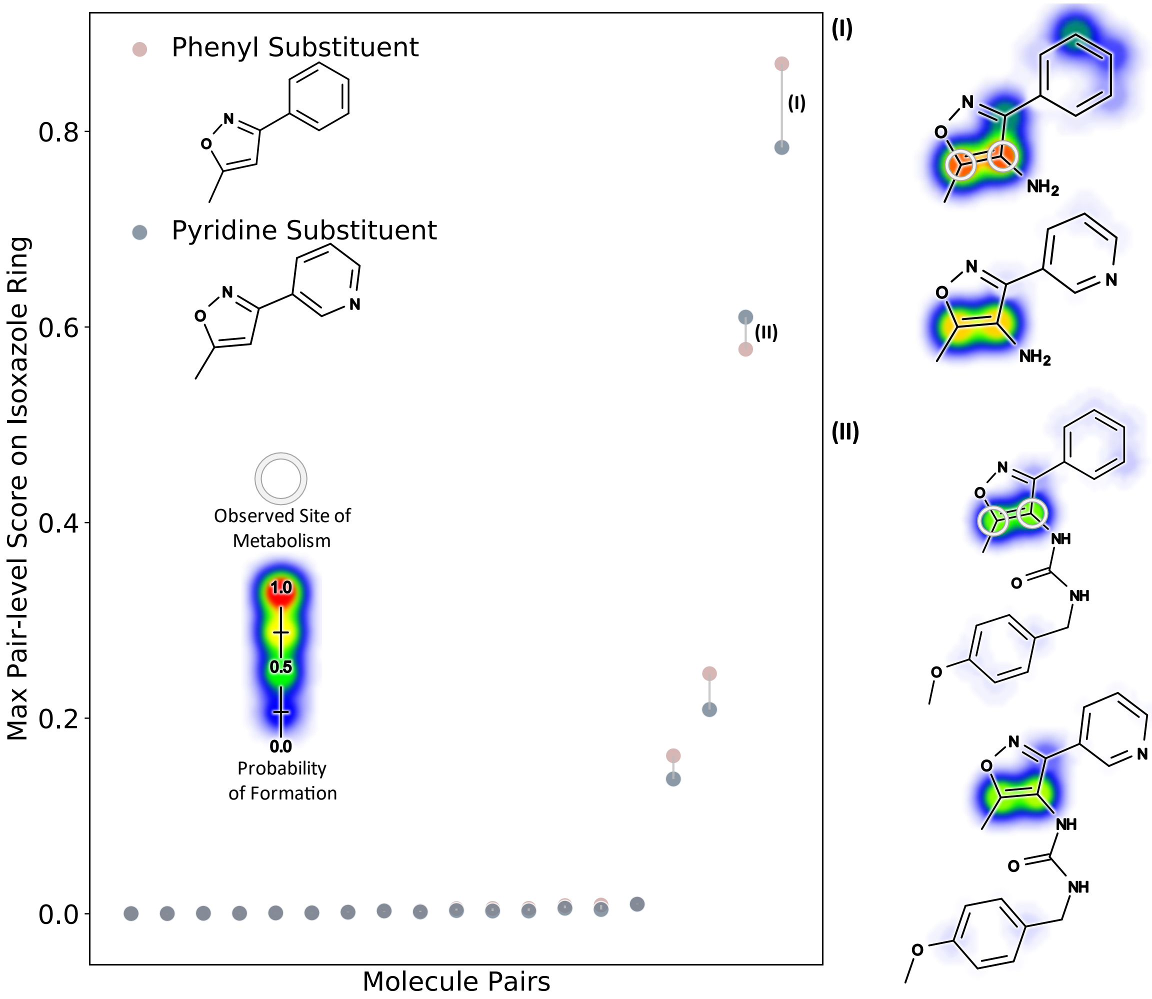

Fig. S5. **Model bioactivation predictions for isoxazoles with either a phenyl or pyridine substituent.** The model predictions follow a general trend of decreasing magnitude when molecules with the phenyl isoxazole are altered to a pyridine isoxazole. There is a distinguishable gap between the two high-scoring pairs, which are the only two pairs observed to form a conjugated electrophile, from the remaining pairs which have lower scores. We collected pairs of molecules based on a common shared structure other than a specific change in substituents between the molecules in a pair. The quinone formation model was applied to the molecule pairs and the maximum atom pair-level predictions involving the isoxazole ring were plotted to check whether the model can differentiate the impact of the substituent as a function of the remaining, shared structure of the pair of molecules. Two molecule pairs are shown to highlight the impact on the model's preferred sites. Pair (I) marks the pair with the most extreme decrease and pair (II) marks the only case where the difference in magnitude increased. For pair (I), the site scores at the 4 and 5 positions of the isoxazole substructure decreased from high to moderate and all sites with any formation potential on the phenyl ring decreased to have no formation potential on the pyridine ring. For pair (II), while the max atom-pair did increase, we still see the expected decrease in magnitude of site-specific predictions in the area localized to the phenyl (now pyridine) ring.

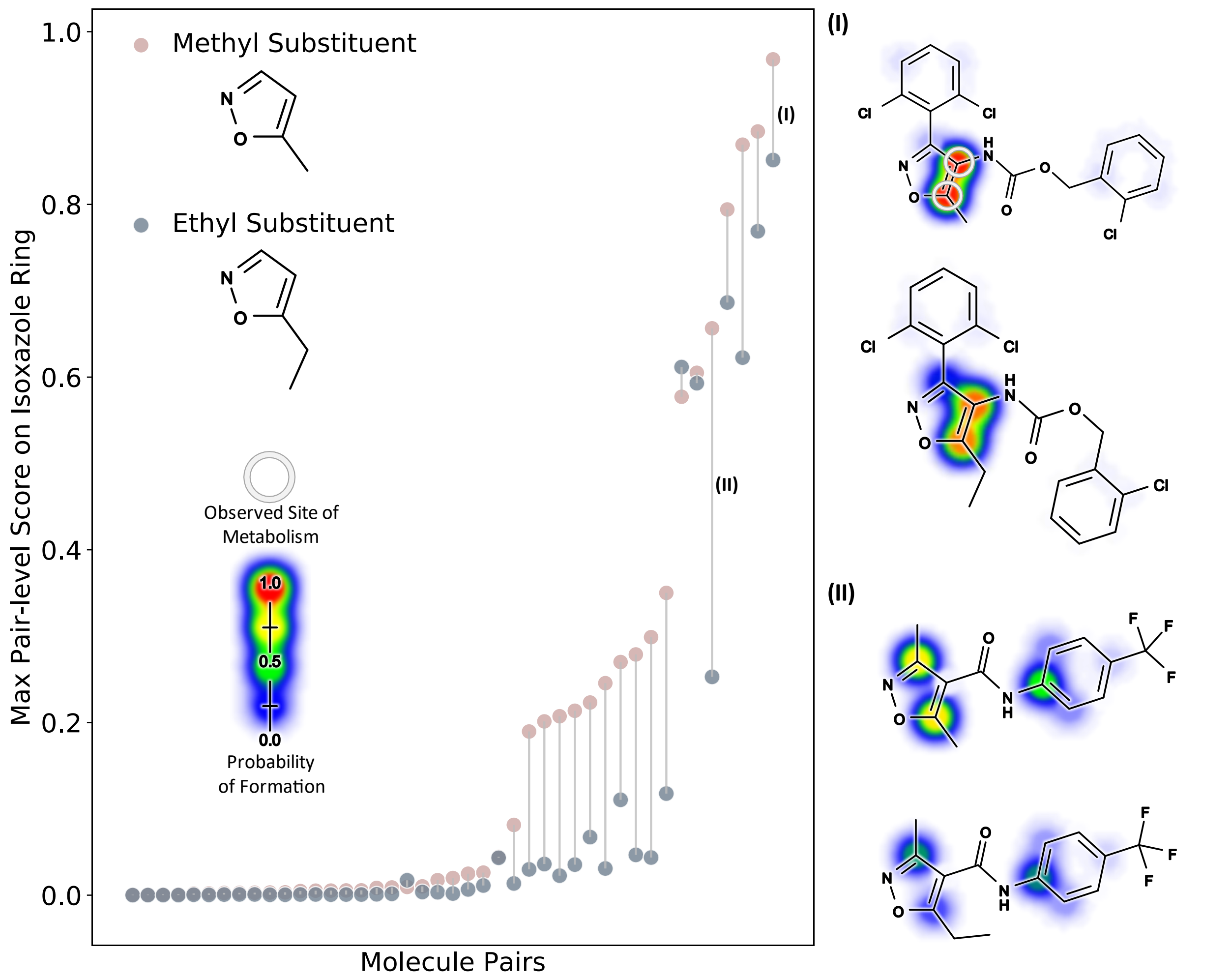

Fig. S6. Model bioactivation predictions for isoxazoles with either a methyl or ethyl substituent. The model predictions follow a general trend of decreasing magnitude when molecules with the 5-methyl isoxazole are altered to a 5-ethyl isoxazole. There is a smooth dynamic range over the magnitudes of the of maximum atom-pair scores and the observed trend is definitive. We collected pairs of molecules based on a common shared structure other than a specific change in substituents between the molecules in a pair. The quinone model was applied to the molecule pairs and the maximum atom pair-level predictions involving the isoxazole ring were plotted to check whether the model can differentiate the impact of the substituent as a function of the remaining, shared structure of the pair of molecules. Two molecule pairs are shown to highlight the impact on the model's preferred sites. Pair (I) is representative of the site-specific changes in model predictions, whereby the altered 5-ethyl causes a decrease in model predictions at the 4 and 5 positions of the isoxazole substructure relative to the original 5-methyl. Pair (II) showcases the most extreme change in the model predictions across all the plotted pairs, with the substituent modification not modifying the specificity of possible sites of reaction but lowering the formation score for all the sites.

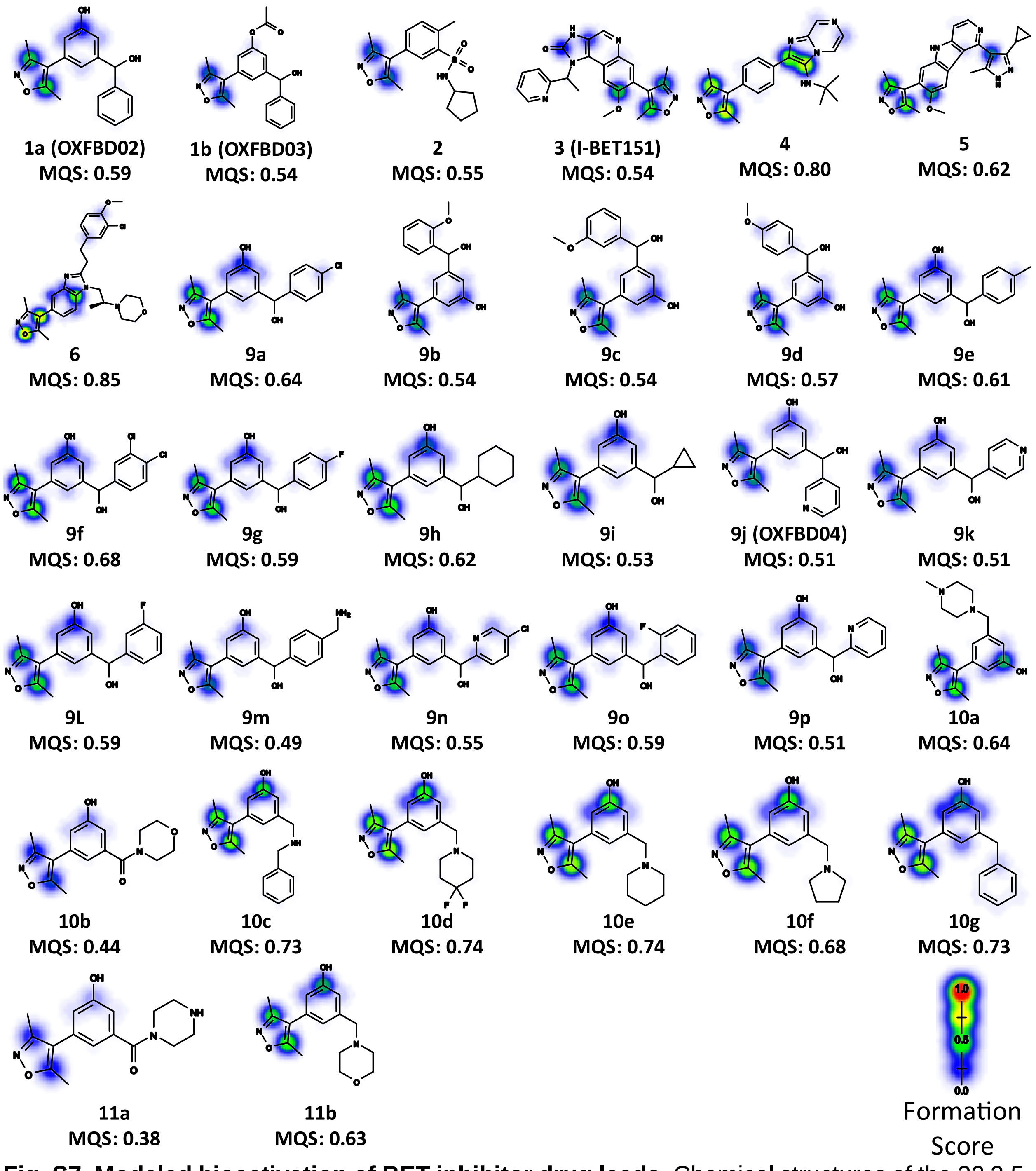

Fig. S7. Modeled bioactivation of BET inhibitor drug leads. Chemical structures of the 32 3,5-dimethylisoxazole-based BET bromodomain ligands reported in Jennings, et al.[47] Molecules overlaid with quinone formation model site-level predictions and include molecule-level scores (MQS).

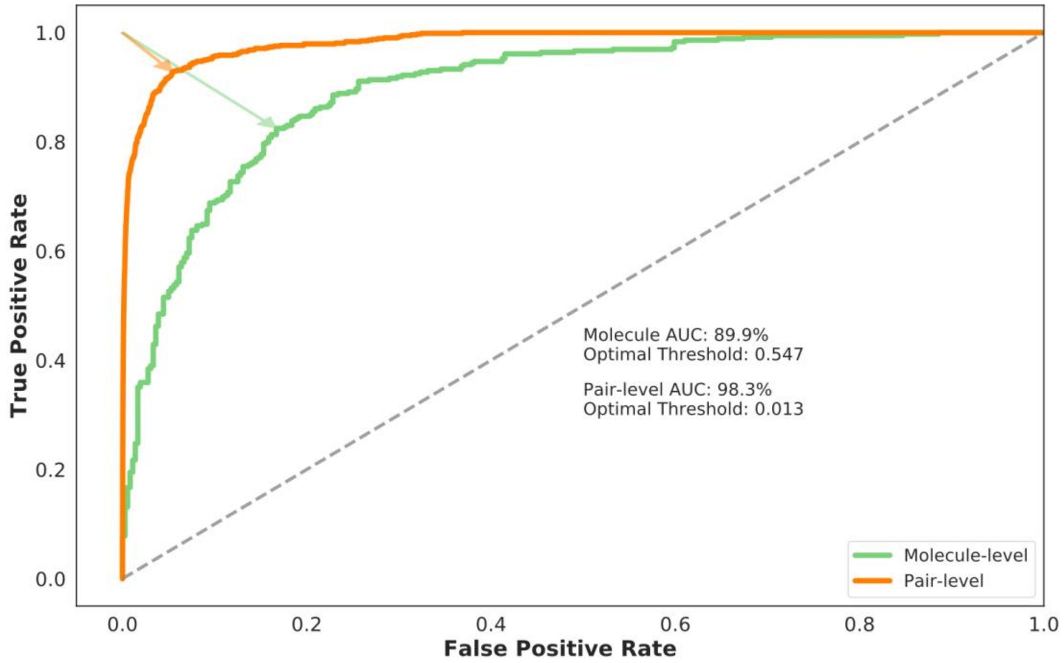

Fig. S8. **Model accuracy analysis for molecule- and pair-level scores for predicted conjugated electrophiles.** The model was validated against 718 molecules with 359 of the molecules observed for form conjugated electrophiles. The molecule-level receiver operating characteristic (ROC) curve is displayed across the 718 molecules. The diagonal dashed line indicates performance that achieves 50% area under the ROC curve (AUC). The arrow points to the location on the ROC curve that offers the optimal tradeoff between sensitivity and specificity. The optimal cutoff for binarizing molecule-level predictions is 0.547. The light green arrow points out the corresponding location of the optimal molecule-level score cutoff on the ROC curve. The optimal cutoff for binarizing pair-level predictions is 0.013. The light orange arrow points out the corresponding location of the optimal pair-level score cutoff on the ROC curve.

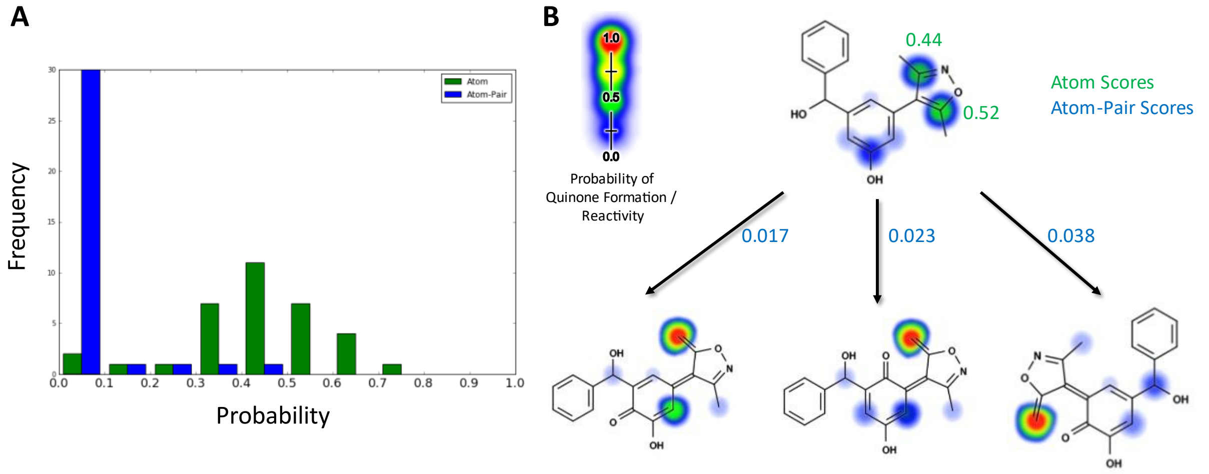

Fig. S9. **Quinone model atom pair and atom scores are different. [A]** (A) We limited the histogram's underlying data to sites that correspond to valid quinone metabolite structures. The atom-pair scores are more reliable and are often lower than the atom scores. **[B]** The two high probability atoms of the parent molecule cannot form a quinone together, so quinone formation is unlikely. However, the highest atom-pair scores correspond to pairs of sites that can form a quinone together, as judged by the structure inference model.

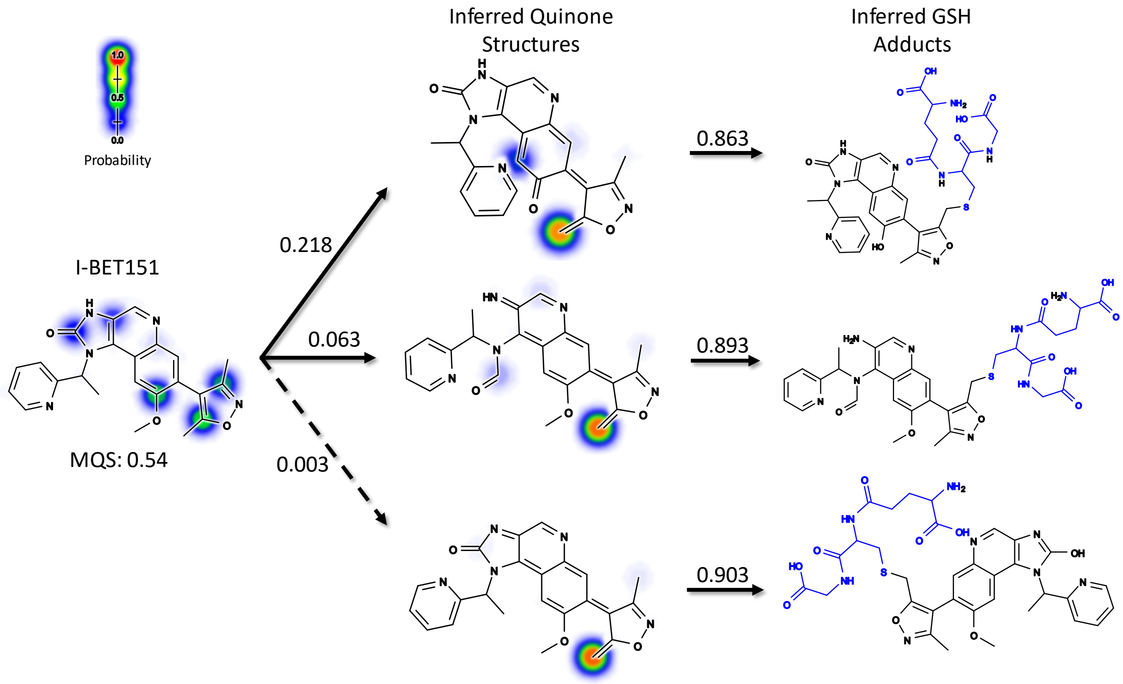

Fig. S10. **Modeled I-BET151 bioactivation pathways.** Top three pathways led only to extended quinone-methides that are shown ranked based on the quinone model score. The dashed arrow denotes a transformation whose prediction was below the model's binarization threshold. All three extended quinone-methides are highly reactive to glutathione leading to the indicated adducts.

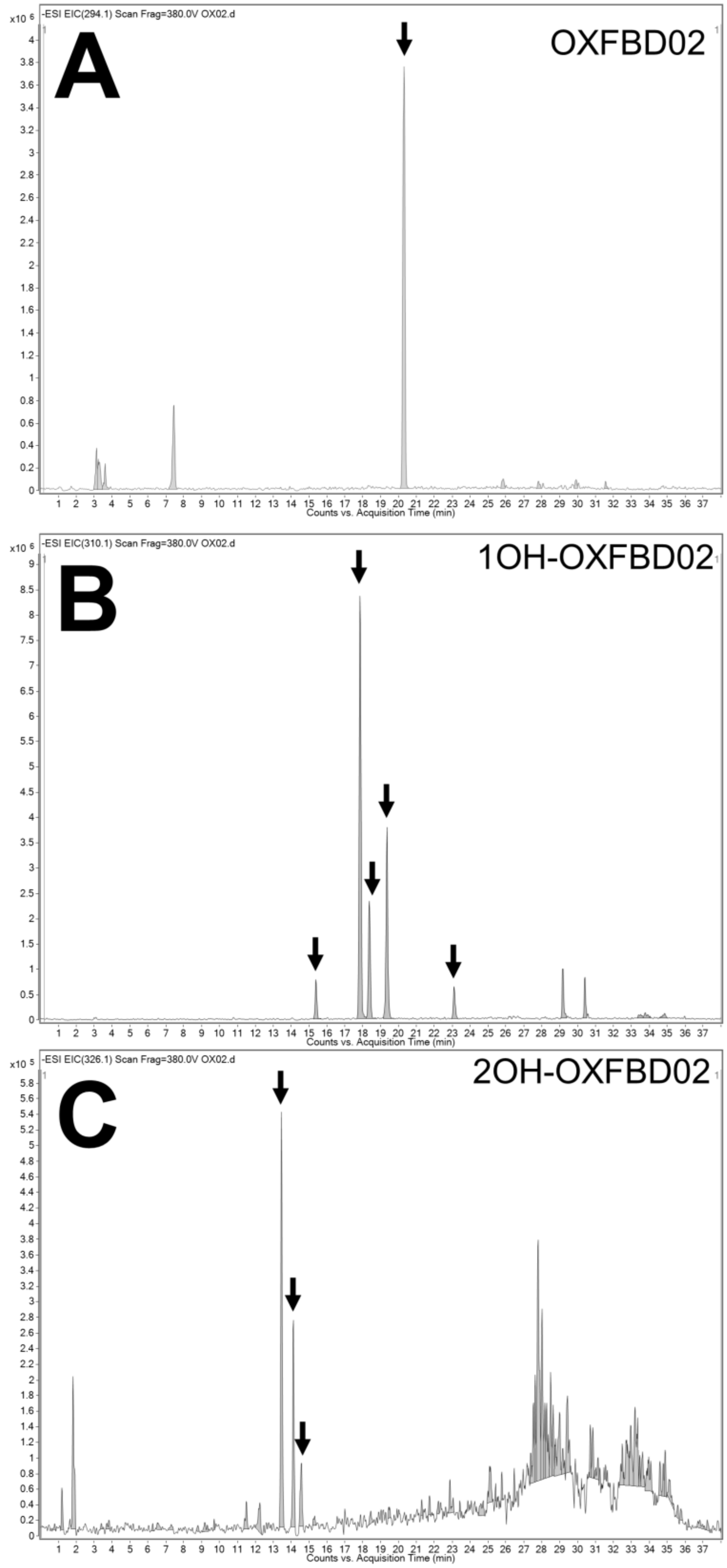

Fig. S11. Mass spectroscopic analysis of OXFBD02 microsomal reactions. Initial total ion scans indicated OXFBD02 (295 *m/z*, **[A]**) oxidation into five mono-hydroxylated metabolites (311 *m/z*, **[B]**) and three di-hydroxylated metabolites (327 *m/z*, **[C]**).

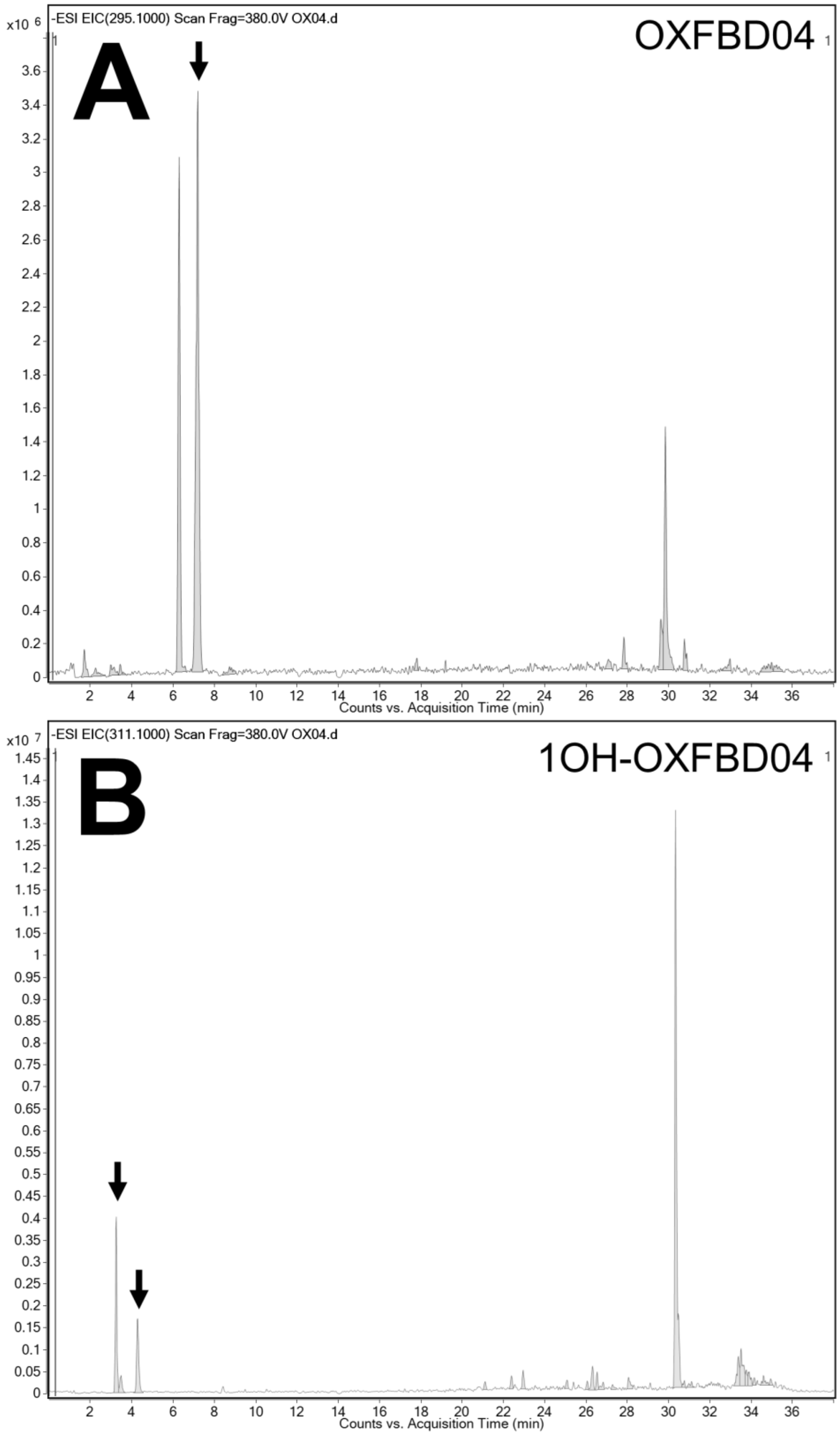

Fig. S12. Mass spectroscopic analysis of OXFBD04 microsomal reactions. Complementary total ion scans showed that OXFBD04 (296 *m/z*, **[A]**) underwent metabolism into only two mono-hydroxylated metabolites (312 *m/z*, **[B]**).

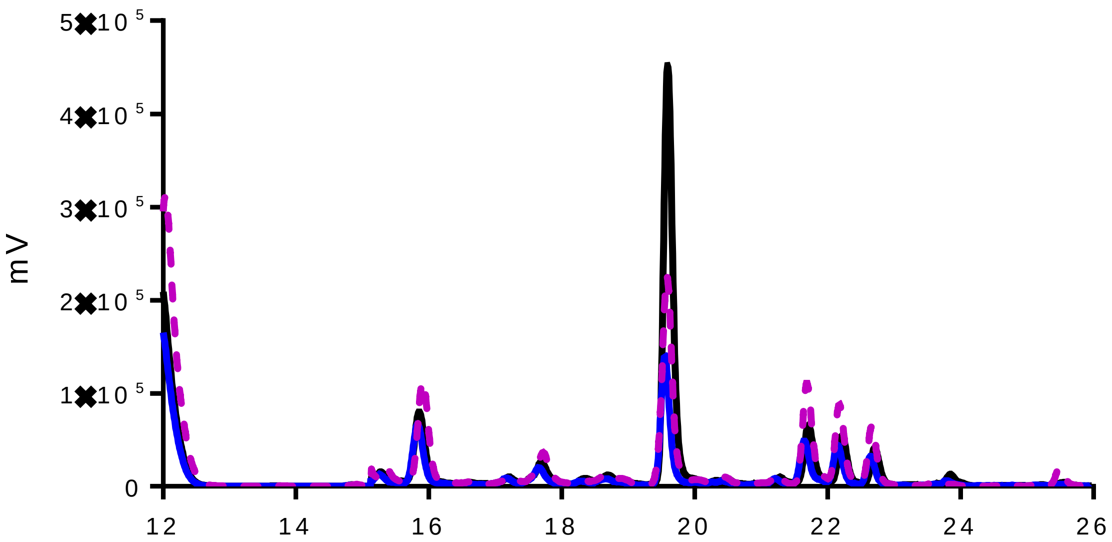

Fig. S13. Fluorescence and MS chromatograms of putative quinone adducts for I-BET151 from human liver microsomal reactions. As observed for OXFBD04, there were no apparent unique fluorescent peaks generated during the metabolism of I-BET151 as a function of time.

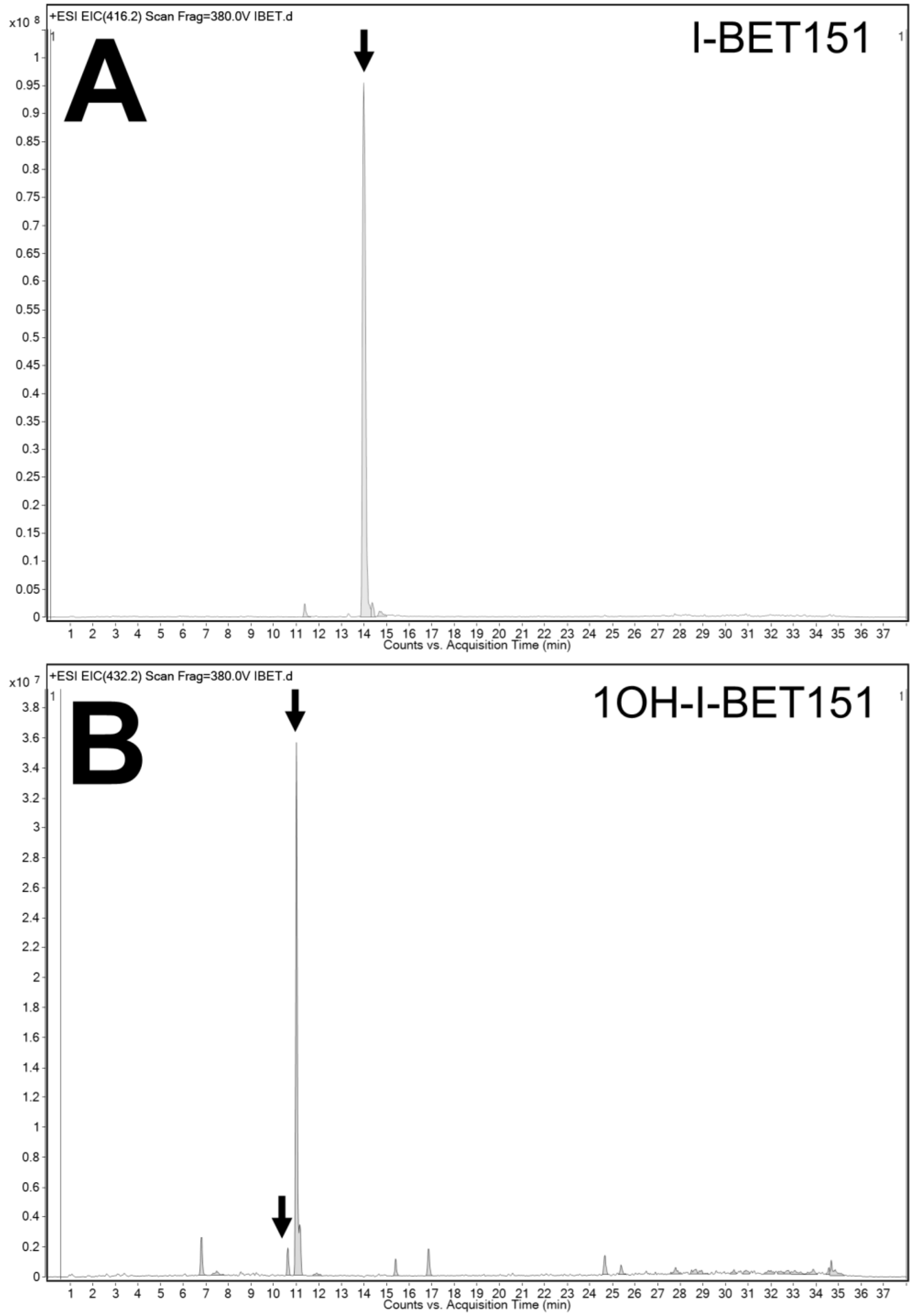

Fig. S14. Mass spectroscopic analysis of I-BET151 microsomal reactions. Subsequent total ion scans showed elution of the parent drug (417 *m/z*, **[A]**) and two mono-hydroxylated metabolites (433 *m/z*, **[B]**), but di-hydroxylated or tri-hydroxylated metabolites were observed.
